# Correlation between nutritional status and oral health quality of life, self-efficacy of older inpatients and the influencing factors

**DOI:** 10.1186/s12877-022-02830-0

**Published:** 2022-04-05

**Authors:** Zhu Zhu, Jiayun Xu, Ying Lin, Kai Chai, Yiyun Zhou, Runyu Jia, Huijue Ni, Minjun Wu, Cuihong Wen, Yuehua Qiao, Haixia Wang, Wei Luan

**Affiliations:** 1 Department of Nursing, Renji Hospital, School of Medicine, Shanghai Jiaotong University, NO.160 Pujian Rd., Shanghai, 200127 China; 2 Department of VIP Service, Renji Hospital, School of Medicine, Shanghai Jiaotong University, NO.160 Pujian Rd., Shanghai, 200127 China

**Keywords:** Older patients, Nutritional status, Oral health quality of life, Malnutrition risk, Self-efficacy

## Abstract

**Objective:**

This study explores the relationship between nutritional status and oral health quality of life, the self-efficacy of older inpatients and the correlative factors.

**Methods:**

In this study, the convenience sampling method was used to select 307 older inpatients in the southern section of the Renji Hospital affiliated to Shanghai Jiao Tong University School of Medicine from October to December 2020 as the main research participants. A mini nutritional assessment questionnaire was used to assess nutritional status, and the Chinese version of a geriatric oral health assessment index questionnaire was used to determine the oral health quality of life. Self-efficacy was assessed by a general self-efficacy scale questionnaire. Descriptive statistics were used to analyse data using the SPSS 22.0 software. Pearson correlation and multiple linear regression analysis were applied to explore the correlation between variables and factors concerned with nutritional status, respectively.

**Results:**

The results of this study showed that the self-efficacy and oral health quality of life of older inpatients were at a moderate level. Among the patients, 263 had one or more tooth defects, and only 128 had oral restorations or wore dentures. The risk of malnutrition in hospitalised older patients was 37.1%, and the incidence of malnutrition was 13.4%. The risk factors of nutritional status of older patients were age, oral-related quality of life, prealbumin index, self-efficacy, chronic disease, monthly income and tooth defect (*P* < 0.05).

**Conclusion:**

The incidence of malnutrition and malnutrition risk in hospitalised older patients is relatively high. The main associated factors include age, tooth defect, oral health quality of life, self-efficacy, chronic disease status and monthly income. Therefore, older inpatients, especially those with prosthodontic problems, should carry out nutritional assessments, intervention and graded management as soon as possible to improve their self-efficacy, improve their nutrition and health status and reduce the incidence of a poor prognosis.

## Introduction

Nutritional status is closely related to the clinical outcome of older inpatients. In 2012, a study by the Chinese Medical Association found that about 50.1% of older inpatients had a nutritional risk, and 15.1% experienced malnutrition [1]. Early detection of malnutrition and timely intervention can improve the health status of most of the elderly [2]. Poor oral hygiene, dental caries, periodontal disease or inappropriate dentures are common oral problems among the elderly [3], and poor masticatory function and oral health are considered risk factors for malnutrition [4]. Self-efficacy refers to the ability of patients to adopt corresponding healthcare behaviours in order to improve indicators and maintain proper health [5]. Improving self-efficacy can effectively enhance oral healthcare awareness and promote good oral healthcare behaviours.

A multitude of physical, social, psychological and biological factors contribute to a person’s nutritional health status. Almost all these factors are particularly pertinent among older adults. Factors such as poverty, oral health behaviour, poor oral hygiene, dental caries and periodontal disease can affect the general and oral health of a person. Previous studies showed that impaired dental status can cause dietary limitations through chewing difficulty, resulting in impaired nutritional status [6]. Furthermore, some reports showed that self-efficacy also affects the nutritional behaviours of the elderly [7].

At present, studies pay more attention to oral health behaviour, oral health-related quality of life of the older or oral health self-efficacy of patients with diabetic periodontitis [8–10]. There are few reports on the correlation between oral health quality of life and the nutritional status of older inpatients [11, 12].

Therefore, this study intends to explore the correlation between nutritional status and oral health quality of life, self-efficacy of older inpatients and the related factors.

## Materials and methods

### Research participants

In this study, the convenience sampling method was used to select 307 older inpatients from the south part of the Renji Hospital affiliated to the Shanghai Jiao Tong University School of Medicine from October to December 2020 as the main research participants. A questionnaire survey was conducted using the Chinese version of a geriatric oral health evaluation index, a mini nutritional assessment and a general self-efficacy scale for the older people. This study complies with the Declaration of Helsinki of the World Medical Association and has been approved by the ethics committee of our hospital (RA-2021-529). All patients signed an informed consent form.

### Inclusion and exclusion criteria

The inclusion criteria were as follows: (1) hospitalised older patients over 60 years of age; (2) those who could eat by mouth and were in a stable condition, understood the content and purpose of the survey, and had no obstacles in communication, reading and writing; (3) those who voluntarily participated in the survey and signed the informed consent form.

The exclusion criteria were as follows: (1) older patients at the end-stage of various diseases; (2) older patients with mental diseases; (3) older patients without autonomous capacity; (4) family members or the patients did not cooperate with the investigation or communication.

### Research methods

Before the questionnaires were issued in this study, the research investigators were uniformly trained. The researcher introduced in detail the purpose and method for filling out the questionnaire to the investigators, then issued the requirements of survey standardisation, standardised operation mode and supervision system in order to ensure the code of conduct and guidelines were implemented. After the training, the investigators were assessed one-to-one. Only those who passed the assessment could start the research work. The investigators collected the general information and questionnaires of the included patients in person. The surveys were conducted after obtaining the informed consent of the respondents, and the respondents could complete the questionnaire independently. If the respondent could not answer independently, the survey was completed with the assistance of the investigator and the questionnaire was withdrawn on the spot.

### Main observation indicators

The main observation indicators of this study included the following three parts: ①Patient’s basic information: patient’s name, hospitalisation number, medical department, gender, age, income, insurance type, household registration location, chronic disease prevalence (according to the Chinese expert consensus on prevention and treatment of chronic diseases, the chronic diseases in this study were defined as patients with cardiovascular and cerebrovascular diseases, cancer, diabetes and chronic respiratory diseases); ② Information about surgery and laboratory indicators: type and grade of surgery, the most recent prealbumin and albumin values in biochemical indicators; ③Oral denture restoration: whether there were dentures and their types, such as movable bridges, movable dentures, etc.

### The Chinese Geriatric Oral Health Assessment Index (GOHAI)

The GOHAI score scale used in this study was developed by Atchison et al. at the University of California in 1990 [13], and it is currently the most commonly used measurement tool for evaluating the oral health-related quality of life of the older people in the world. The Chinese version of the GOHAI was translated by Ling Junqi et al. [14] The scale has four dimensions and 12 items, involving physical function, psychosocial function, pain and discomfort. The Likert 5-level scoring method is used, and the scores are as follows: never = 1 point, rarely = 2 points, occasionally = 3 points, often = 4 points, frequently = 5 points. Items 3, 7 and 11 are scored by reverse scoring. The total score of the scale is 60; the higher the score, the worse the oral health quality of life. The scale has good reliability and validity. The Cronbach’s α coefficient is 0.81.

### Mini Nutritional Assessment (MNA) for the older people

The MNA score table was first proposed by Guigoz et al. [15] in 1996. It has been widely used abroad and has good reliability and validity. MNA is composed of 18 indicators in four parts: anthropometric indicators, overall assessment, diet assessment and subjective assessment. The total score is 30 points. The scoring standard is as follows: 24–30 points indicate good nutrition; 23.5–17.0 points have the risk of malnutrition; 17.0 points or fewer indicate malnutrition. MNA is recommended internationally as a screening tool for malnutrition in older inpatients [16]. MNA has high sensitivity (97.9–100%) and specificity (69.5–100%) [17].

### General Self-Efficacy Scale (GSES)

The Chinese version of the GSES was first established by Zhang Jianxin and Schwarzer et al. in 1995 [18]. There are 10 items in total, involving the individual’s self-confidence when encountering setbacks or difficulties. The Likert 4-level scoring method is used, and each item is scored from 1 to 4. For each item, the subjects answer ‘completely incorrect’, ‘somewhat correct’, ‘mostly correct’ or ‘completely correct’ according to their actual situation. The score is divided into three levels: 31–40 is good, 20–30 is medium and 10–19 is low. The higher the score, the better the self-efficacy. The scale has good reliability and validity. The Cronbach’s α coefficient is 0.87.

### Multiple regression analysis

In this study, the result of single factor analysis was used as a reference, and 11 types of variables were assigned respectively: X1 = age, X2 = household registration location, X3 = monthly income, X4 = chronic disease, X5 = whether they have received health education about oral health knowledge, X6 = albumin value, X7 = prealbumin value, X8 = tooth defect, X9 = oral restoration or denture, X10 = oral health quality of life score classification, X11 = general self-efficacy score classification. The total score of the MNA was used as the dependent variable to perform stepwise regression analysis. All the data were entered into the SPSS 22.0 statistical software, and the stepwise regression method was used to construct the model. = 0.15, and The independent variable collinearity calculation was performed.

### Statistical methods

In this study, the SPSS 22.0 statistical software was used for data processing. The results of the questionnaire were entered into an Excel table by two persons, and continuous variables were presented as means and standard deviations. The counting data were expressed as percentages (%). A t-test or one-way analysis of variance (ANOVA) was used for comparison between groups with normal distribution, and a nonparametric test was used for comparison between groups without normal distribution. The Chi-square test was used for the counting data. Pearson correlation analysis was used to analyse the correlation between variables, and multiple linear regression analysis was used to analyse the related factors of nutritional assessment results of older inpatients. A *P* value of < 0.05 was statistically significant.

## Results

### General information

In this study, 320 patient questionnaires were issued, and 307 valid questionnaires were returned. The effective recovery rate was 95.9%. Among the older patients surveyed in this study, 172 were males and 135 were females, with an average age of 71.192 ± 8.835 years old. The places of household registration were 101 in the city, 87 in the urban–rural area, and 119 in the rural area. The education level was mainly junior high school and below (78.5%). Nearly one-third of the participants had a monthly income of 2500–5000 yuan/month and all of them had medical insurance of different types. Among the patients, 60.5% suffered from one or more chronic diseases, 259 had never received health education about oral health knowledge and 263 had one or more tooth defects, of which 128 had undergone oral restoration or wore dentures.

### Oral healthcare, nutrition evaluation and self-efficacy scale scores of older inpatients

The results of this study showed that the general self-efficacy scores, the total scores of oral health quality of life and the total nutritional evaluation scores of surveyed older patients were at a moderate level. The MNA scores of 152 cases ranged from 24 to 30, indicating good nutrition; 114 cases had a score of 23.5–17.0, which indicated a risk of malnutrition; 41 cases with a score of less than 17 were at risk of malnutrition. The incidence of malnutrition risk in this study was 37.1%, and the incidence of malnutrition was 13.4%. See Table 1 for details.

**Table 1:** Scores of oral health care, nutrition evaluation and self-efficacy scale

Indicator	GSES score	GOHAI score	MNA score
Average	25.426	27.969	27.664
Standard deviation	6.774	7.887	5.048
Minimum	10.00	12.00	3.00
Maximum	40.00	51.00	36.00

### Correlation of oral healthcare, nutritional evaluation and self-efficacy scale scores of older inpatients

The results of this study showed that the simple nutritional evaluation and general self-efficacy of older hospitalised patients exhibited a significant positive correlation (*P* < 0.01), and the oral health quality of life exhibited a significant negative correlation with general self-efficacy and simple nutritional evaluation (*P* < 0.01). See Table 2 for details.

**Table 2:** Correlation of oral health care, nutritional evaluation and self-efficacy scale scores in hospitalized elderly

Indicator		MNA score	GOHAI score	GSES score
MNA score	Pearson related	1		
	Correlation (two-tailed)	/		
GOHAI score	Pearson related	-0.407^**^	1	
	Correlation (two-tailed)	0.000	/	
GSES score	Pearson related	0.206^**^	-0.233^**^	1
	Correlation (two-tailed)	0.000	0.000	/

**The correlation is significant at the 0.01 level (two-tailed)

### Single-factor analysis of the nutritional assessment of older inpatients

The results of this study showed that the different demographic factors of the research participants had been tested to be normally distributed, and the scores of each scale were also normally distributed. Therefore, the above items were used as independent variables (the distribution of each scale is divided into corresponding levels by scores) and the simple nutrition evaluation scores were used as the dependent variable for single-factor analysis. The results of the study showed that there were significant differences in the average score of nutritional assessment in terms of the 11 variables (*P* < 0.05). See Table 3 for details.

**Table 3:** Single factor analysis of mini-nutritional assessment scale for elderly patients

Item		Number of cases	($\overline x$ ±S)	F/t value	***P*** value
**Gender**	Male	172	21.837**±**4.647	0.616	0.433
	Female	135	21.381**±**5.523		
**Department**	Surgical Department	178	21.134**±**5.140	2.609	0.075
	Internal Medicine Department	129	22.408**±**4.868		
**Age**	61-70 years old	173	22.482**±**4.295	6.022	0.001^*^
	71-80 years old	82	21.396**±**4.811		
	81-90 years old	44	19.284**±**6.908		
	91 years old and above	8	18.750**±**6.065		
**Education level**	Primary school and below	122	21.127**±**5.354	1.697	0.618
	Junior high school	119	21.979**±**4.580		
	Senior high school and technical secondary school	38	21.039**±**5.932		
	College and above	28	23.214**±**3.952		
**Insurance type**	Urban and rural resident insurance	244	21.629**±**5.152	0.773	0.462
	Four types of insured medical insurance	34	22.397**±**4.432		
	Non-local medical insurance	29	20.810**±**4.850		
**Registered permanent residence**	Urban area	101	23.109**±**3.736	14.081	0.000^**^
	Urban-rural area	87	21.648**±**4.875		
	Rural area	119	16.250**±**7.408		
**Monthly economic income**	Less than 2500 yuan/month	68	18.67**±**66.025	14.675	0.000^**^
	2500-5000 yuan/month	100	19.600**±**4.992		
	5001-8000 yuan/month	89	22.250**±**3.023		
	More than 8001 yuan/month	29	23.363**±**3.256		
**Chronic disease**	None	121	22.632**±**4.372	3.022	0.030^*^
	1 type	98	21.331**±**5.289		
	2 types	73	20.506**±**5.320		
	3 types and above	15	20.100**±**6.121		
**Have you received health education about oral health knowledge?**	Yes	47	22.000**±**4.717	2.173	0.046^*^
	Never	259	20.583**±**5.088		
**Have you had any surgery and the level of surgery?**	None	109	21.674**±**5.072	1.222	0.301
	Level 1	32	23.062**±**3.468		
	Level 2	71	21.162**±**5.753		
	Level 3	50	20.770**±**5.573		
	Level 4	39	22.051**±**3.906		
**Albumin grading**	Abnormal	135	19.800**±**5.927	35.499	0.000^**^
	Normal	172	23.078**±**3.648		
**Prealbumin grading**	Abnormal	126	19.337**±**5.865	51.692	0.000^**^
	Normal	181	23.237**±**3.626		
**Tooth defect condition**	No defect	44	21.710**±**4.933	5.760	0.015^*^
	One or more teeth defects	263	20.744**±**3.359		
**Oral restoration or denture condition**	None	179	21.664**±**5.284	2.430	0.026^*^
	One fixed bridge	26	23.884**±**3.207		
	Two or more fixed bridges	26	18.826**±**4.890		
	Partial removable denture	30	21.366**±**5.396		
	Fixed bridge + partial movable denture	3	21.166**±**4.310		
	Complete denture	30	21.716**±**4.563		
	Implanted teeth	13	22.923**±**3.232		
**Oral health quality of life score classification**	Good	64	23.109**±**3.736	14.081	0.000^**^
	Medium	225	21.648**±**4.875		
	Bad	18	16.250**±**7.408		
**General self-efficacy score classification**	Bad	41	21.122**±**5.934	3.261	0.040^*^
	Medium	205	21.304**±**4.954		
	Good	61	23.098**±**4.506		

^**^The correlation is significant at the 0.01 level; ^*^The correlation is significant at the 0.05 level

### Multiple regression analysis of relative factors on nutritional assessment of older inpatients

After a total of eight variable iteration screenings, the research results showed that a total of eight factors were included in the variable equation model. The model was well constructed and had a certain predictive effect on MNA. The summary of the regression equation model and analysis results are shown in Table 4.

**Table 4:** Multiple regression analysis on Influencing Factors of simple nutritional assessment score for the elderly

Model		Nonstandard coefficient		Standardization coefficient	T	Sig.	Collinearity statistics	
		B	Standard error	Beta			Tolerance	VIF
8	(constant)	18.855	2.965		6.359	0.000		
	Age	-0.081	0.028	-0.142	-2.870	0.004	0.932	1.073
	Oral quality of life	-2.625	0.489	-0.258	-5.371	0.000	0.987	1.014
	General self efficacy	1.664	0.416	0.204	3.999	0.000	0.878	1.139
	Chronic basic diseases	-0.799	0.274	-0.143	-2.916	0.004	0.955	1.047
	Monthly economic income	1.553	0.478	0.160	3.248	0.001	0.944	1.060
	Tooth defect	-1.623	0.706	-0.113	-2.301	0.022	0.954	1.048
	Albumin index	2.374	0.644	0.231	3.686	0.000	0.583	1.716
	Prealbumin index	1.365	0.652	0.134	2.094	0.037	0.559	1.790

*R*=0.579, *R*^2^=0.336, After adjustment *R*^2^=0.317, F=18.374, *P*<0.001

## Discussion

Appropriate and adequate nutrition of the older people is of great importance for their general and oral health. Diet plays an important role in preventing disease in the elderly [19]. It has been shown that general health and quality of diet are determined by social support, socioeconomic status, culture and oral health [20]. In the present study, the single factor and multiple regression analysis were performed, through which the understanding of the relationship among nutritional status, oral health quality of life and self-efficacy of older inpatients is deepened.

The level of average self-efficacy and average oral health quality of life of this study are consistent with previous research conclusions [5]. In terms of oral restoration, there were problems such as a high incidence of dentition defects and a low restoration rate, as well as a lack of education about oral healthcare, which is similar to the results of Wang Zhonghua’s research [21]. In the results of this study, the incidence of malnutrition and malnutrition risk were lower than the results of the 2012 national nutrition survey of older inpatients [1], which relates to the rise in the overall economic level of our society, the improvement in the living standards of residents and the rich social support, as evidenced by the surveyed area being located in a very large city. As the immune function of the elderly is relatively reduced, accompanying malnutrition can increase the chance of infection, leading to an increase in hospitalisation days, hospitalisation costs and mortality [22], which requires giving this matter full attention.

The results of this study show that the oral health awareness and self-efficacy level of older inpatients might be related to their nutritional status and enhancing the oral health quality of life and self-efficacy of older patients will help improve their nutritional status. Previous studies have shown that malnutrition is an important cause of the decline in the quality of life of the elderly; it increases the incidence of chronic diseases and weakness, and it creates obstacles to self-care ability, which can lead to an increase in the economic burden of society and families [23]. Medical staff should actively help the older people establish a good oral healthcare concept, assist in changing their bad eating habits and increase self-efficacy. Meanwhile, oral health education is also significant in establishing the quality of life.

This study found that the patient’s age, oral-related quality of life, albumin index, prealbumin index, self-efficacy, chronic disease, monthly income and tooth defects were risk predictors of the nutritional status of older inpatients. The older the patient, the worse the economic situation, and the corresponding level of oral-related quality of life and nutritional status may be reduced. The nutritional status of patients with fewer basic diseases may be better than that of patients with more basic diseases. The condition of albumin and prealbumin can reflect the protein status of the human internal organs and can better reflect the storage and consumption of nutrients [24, 25]. Malnutrition is a related factor for the poor prognosis of many chronic diseases. Early detection of malnutrition and timely intervention can improve the health of the vast majority of the elderly and reduce the incidence of chronic diseases [26]. It is suggested that in clinical work, medical staff should carry out early nutritional assessments, conduct intervention and graded management for older patients with advanced age, and evaluate for tooth defects, chronic diseases, low monthly income and low self-efficacy level, so as to improve the patients’ self-efficacy, nutrition and health status, thereby reducing the incidence of a poor prognosis.

In 2016, a systematic review divided the risk factors that can lead to malnutrition in older patients into five categories: physical function, mental state, oral hygiene, social factors and eating behaviour [27]. It can be seen that improving the nutritional status of the older people requires comprehensive consideration from multiple aspects. In addition, older patients are susceptible to diseases, their physiological and psychological adaptability are reduced during hospitalisation and risk factors for malnutrition are more and more complex. Therefore, it is necessary to evaluate and intervene correctly in light of the different conditions of older hospitalised patients through early identification of relevant risk factors and biochemical indicators, timely nutritional intervention or nutritional support treatment for the older people who have malnutrition or malnutrition risk. At different times of in-hospital nursing or extended nursing, targeted health guidance and health education should be adopted according to the age, education level and hobbies of the older patients, especially those with oral problems. These practices will guide them in regularly screening their oral and nutritional status, choosing the correct behaviour of eating, enhancing their awareness of oral healthcare and ultimately improving their nutritional and health status, reducing the incidence of a poor prognosis.

This research still has shortcomings. First of all, this study is a single-centre clinical study, and subsequent multicentre clinical studies are still needed for further discussion. Secondly, the sample size included in this study is relatively small, and it is still necessary to increase the sample size for further research.

## Conclusion

The main related factors of nutritional status include age, tooth defect, oral health quality of life, self-efficacy, chronic disease and monthly income, etc. Therefore, older inpatients, especially those with oral restoration problems, should have nutritional assessment, intervention and graded management carried out as soon as possible to improve their self-efficacy, improve their nutrition and health status and reduce the incidence of a poor prognosis.

## Data Availability

The datasets used and analyzed during the current study are available from the corresponding author on reasonable request.

## Abbreviations

GOHAI: Geriatric Oral Health Assessment Index
MNA: Mini-nutritional Assessment
GSES: General Self-Efficacy Scale
